# Screening for Cytomegalovirus during Pregnancy

**DOI:** 10.1155/2011/942937

**Published:** 2011-08-09

**Authors:** Stuart P. Adler

**Affiliations:** Medical College of Virginia Campus, Virginia Commonwealth University, P.O. Box 980163, Richmond, VA 23298-0163, USA

## Abstract

The epidemiology and pathogenesis of CMV infections among pregnant women have been intensely studied over the last three decades. This paper highlights recent developments that make either universal or limited serologic screening for CMV during pregnancy potentially attractive. The developments include an understanding of the pathogenesis of CMV infections, a knowledge of high-risk women, the availability of accurate methods for the serologic diagnosis of a primary CMV infection using either single or serial blood samples, accurate methods for the diagnosis of fetal infection via amniotic fluid, sensitive fetal and placental indicators for neonatal outcomes, and the availability of potentially effective interventions.

## 1. Introduction

The epidemiology and pathogenesis of CMV infections among the people of the USA and particularly among pregnant women have been intensely studied over the last three decades [1, 2]. We know that a primary CMV infection during pregnancy is a frequent and serious threat to the fetuses of pregnant women. Each year in the USA, an estimated 40,000 pregnant women acquire a primary CMV infection (seroconvert) during pregnancy. Of the 40,000 women who seroconvert approximately 6,000 to 8,000 of their infants will develop severe and permanent neurologic damage from this infection [3]. Another less frequent effect is fetal death or neonatal death which occurs in about 10% of fetuses or newborns following an intrauterine CMV infection. Neurologic damage includes impaired development, mental retardation, and neurosensory hearing deficit. 

 The rate of susceptibility to CMV during pregnancy is also well established. Among women of child-bearing age between 40% and 80% will be susceptible (seronegative) to CMV at the beginning of pregnancy. The rate of susceptibility at the beginning of pregnancy varies by ethnic or racial group with highest rates occurring among African-American and Hispanic populations [2]. 

In 1999, the Institute of Medicine issued a report on priorities for new vaccines and gave development of a CMV vaccine level-one priority [4]. This was based not only on the frequency of neurologic disease but also on the fact that CMV is the most common cause of nonhereditary hearing loss with an estimated 25 percent of all hearing deficit due to a congenital CMV infection [5]. Further, CMV is a much more common cause of severe neurological damage in infancy than was bacterial meningitis, congenital rubella, or neonatal herpes simplex infections [4]. 

In spite of the detailed knowledge about the epidemiology and pathogenesis of CMV infections in pregnant women, this infection remains largely unknown to the majority of women in the United States [6]. Few, if any, pregnant women are routinely screened for CMV infections during pregnancy. Questions surrounding the appropriateness of serologic screening for CMV during pregnancy are important because over 90% of primary maternal CMV infections during pregnancy are asymptomatic and may remain asymptomatic in the fetus. Israel and eight European countries (France, Belgium, Spain, Italy, Germany, Austria, Portugal, and the Netherlands) routinely screen the majority of pregnant women serologically for CMV [7, 8]. This routine serologic screening occurs without the recommendations or guidelines of any governmental agency, authority, or a professional medical society. 

Routine serologic screening for CMV of pregnant women in Europe has yielded very important advances in our understanding of CMV infections among pregnant women. Near universal testing in Belgium has yielded definitive data concerning maternal-fetal transmission rates of CMV as a function at gestational age [9]. The Italians have capitalized on national serologic screening to develop and evaluate methods to diagnose maternal and fetal CMV infections including the CMV IgG avidity assay, and to test interventions such as CMV immunoglobulin [10, 11]. The French have used serologic screening to evaluate the role of maternal education about CMV and the role of hygienic intervention to prevent maternal acquisition of CMV during pregnancy [12]. 

This paper will highlight recent developments that make either universal or limited serologic screening for CMV during pregnancy potentially attractive. The developments include a much better understanding of the pathogenesis of CMV infections, a knowledge of high-risk women, the availability of accurate methods for the serologic diagnosis of a primary CMV infection using either single or serial blood samples, accurate methods for the diagnosis of fetal infection via amniotic fluid, sensitive fetal and placental indicators for neonatal outcomes, and the availability of potentially effective interventions. 

## 2. Pathogenesis of Congenital CMV Infections

**Figure 1:**
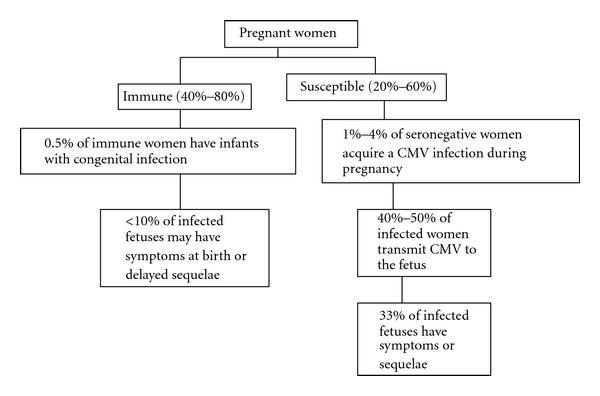
Relationship of maternal immunity to disease caused by congenital CMV infection. Adapted from [19].

Figure 1 shows an algorithm which indicates that between 40% and 60% of pregnant women are susceptible to CMV at conception. Of these, between 1% to 4% will acquire CMV during pregnancy, and on average between 40% and 50% of infected women will transmit the virus to the fetus. The lowest transmission rate (35%) occurs when the maternal infection is in the first trimester, and as pregnancy progresses, the transmission rate increases to 73% for women who acquire CMV infections in the third trimester [9]. Of infants infected *in utero* approximately a third will have symptoms or develop severe neural impairment [11]. This neonatal disease rate is probably highest for children of women who have had a primary infection in the first half of pregnancy, but definitive data on this point are lacking. 

There is considerable uncertainty about the role of maternal immunity to CMV prior to conception. Infants born of mothers with preconception immunity not only give birth to infected infants but also occasionally give birth to infants with symptoms at birth that may develop delayed sequelae, particularly hearing deficit [3, 13]. Nevertheless, there is no uncertainty concerning the fact that the rate of congenital infection among women with preconception immunity is only between 0.5% and 2% as compared to an average of 40% to 50% in women who have seroconverted during pregnancy. In one study, 3% of prepregnant women seronegative at or before conception had congenitally infected infants compared with 1% for women seropositive before conception [14]. 

A 1992 study demonstrated the most severe infant sequelae occurred only among women who had a primary CMV infection during pregnancy [3]. Infant hearing loss was observed in women who had a recurring infection, but it was not nearly as profound as among the children born of mothers without preconception immunity [13]. Hence, eliminating or reducing the morbidity associated with a primary infection during pregnancy is the focus of serologic screening during pregnancy.

In Italy, routine serologic screening for CMV infections among pregnant women has led to an understanding of the effect of antibodies in preventing and/or possibly reversing the viral effects of a primary infection during pregnancy [11, 15–17]. These studies found that the primary effect of antibodies is most likely on the placenta which, during a primary CMV infection in the mother, becomes dysfunctional and results in poor oxygenation and nourishment of the fetus *in utero* [16]. Thus, many symptoms of congenital CMV infection that are present at birth may not be due to any direct effect of the virus on the fetus but rather to the infection of the placenta which impairs its capacity to provide oxygen and nutrition to the developing fetus. Several lines of evidence suggest this possibility. 

First, most manifestations of congenital infection (such as fetal growth retardation, liver disease, hematopoietic abnormalities, and splenomegaly) resolve over the early weeks and months of life, concurrent with adequate oxygenation and nutrition. Second, many infants born to mothers with CMV infection are asymptomatic and develop normally, despite viremia *in utero* and postnatally and viral shedding in urine and saliva for years after birth. Third, CMV infection is occasionally associated with a “blueberry muffin” syndrome, in which purpura is caused by extramedullary haematopoiesis that indicates intrauterine hypoxia. Fourth, hepatomegaly is due to biliary obstruction, secondary to extramedullary haematopoiesis and erythrocytic congestion which is also responsible for splenic enlargement in most symptomatic infants [18]. The increase in placental size occurs with primary maternal CMV infection because the placental vasculature enlarges to compensate the fetus [16]. The beneficial effect of antibodies may be mediated through improved placental function and enhanced supplies of oxygen, substrates, and nutritional elements to the fetus. Finally, it has been recently observed that CMV hyperimmunoglobulin therapy is associated with reduced placental inflammation and size and fetal ultrasound abnormalities [11, 15, 16]. 

## 3. High-Risk Women

The major risk factor for maternal acquisition of CMV during pregnancy is frequent and prolonged contact with a child less than three years of age [20–28]. This occurs among women with a child in the home or among women employed in child care centers [24, 29, 30]. CMV seronegative health care workers, even those caring for hospitalized young children and infants, are not at an increased risk [31]. Regardless of whether CMV was acquired *in utero,* via breast milk, or via contact with other children, unlike older children and adults, children who are less than age 2 years when they acquire CMV, shed CMV in urine and saliva for 6 to 42 months with a median of 18 months [32]. In the USA, 60% of the mothers of children in daycare are CMV seronegative, and at least 25% of all young children attending large group child care centers are shedding CMV. Seronegative mothers with infected children acquire CMV at rates, 10 to 25 times higher than other women in the population [21, 27]. The annual infection rate for seronegative women without exposure to children is 2% [24].

To estimate the frequency of pregnancy and exposure to CMV among mothers contemplating a possible additional pregnancy and with a child less than 2 years of age in group daycare, we recently performed a prospective study which included a demographic questionnaire and serologic and virologic monitoring of mothers and their children in daycare [33]. Of 60 women, 62% were seronegative and 20% of seronegative women had a child shedding CMV. Of the 60 women, 23 women or 38% (95% CI = 0.27, 0.51) became pregnant on average 10 months after study enrollment. During pregnancy, eight or 35% (95% CI = 0.19, 0.55) of these pregnant women had a child in daycare who shed CMV. These results illustrate the potential magnitude of the public problem associated with exposure to a silent viral infection during pregnancy. Our data, when extrapolated to the US population, estimate that every two years between 31,000 and 168,000 susceptible pregnant women will be exposed to CMV by an infected child.

Another group of high-risk women are those who are seronegative, young, poor, and predominantly African-American. Even for this group, contact with a young child is an independent predictor of delivering a CMV congenitally infected infant, as is a history of frequent sexual activity [28]. A recent study monitored CMV seroconversion among 1906 seronegative women attending a fertility clinic [20]. Seroconversion was associated not only with contact with a child younger than age 3 years but also with seropositivity of the sex partner. Thus, it is likely that CMV is transmitted not only via the oral mucosal route, but also via the vaginal mucosal route. Because CMV is often present in semen, it may be prudent to include condom use as part of the hygienic precautions given to seronegative pregnant women. 

## 4. Accurate Methods for Serologic Diagnosis

The gold standard of serologic diagnosis is maternal seroconversion based on the detection of IgG antibodies to CMV. The IgG assay is nearly 100% sensitive and specific, readily available, and automated for high volume capacities [34, 35]. In the absence of universal serial serologic screening of pregnant women, diagnosis via seroconversion is seldom achieved since an initial seronegative serum is rarely available. The detection of IgM antibodies in maternal sera can be helpful but has problems; although IgM antibodies to CMV occur in all primary infections, they may also occur after reactivations or reinfections and the assay has a high false positive rate. We and others have observed that IgM usually peaks 3 to 6 months after a primary infection but may remain present in serum for over 12 months [36]. Hence, finding IgM to CMV in a single serum of a pregnant woman does not alone establish a recent primary CMV infection during pregnancy [37]. 

Antibody avidity, which is an indirect measure of the tightness of antibody binding to its target antigen, increases in the first weeks after a primary infection. Low avidity IgG antibodies to CMV persist for up to 20 weeks after a primary CMV infection [10]. These low avidity antibodies are then replaced by high avidity antibodies (>60% binding in presence of 5 M urea). Currently, the combination of the presence of anti-CMV IgM antibodies and low avidity anti-CMV IgG antibodies along with maternal or fetal symptoms is used for the diagnosis of a primary maternal infection [38]. 

## 5. The Diagnosis of Fetal Infection via Amniotic Fluid

Amniotic fluid is a helpful adjunct in maternal diagnosis but cannot replace maternal serologic testing because amniotic fluid may contain CMV even if the mother was immune to CMV before conception. The best test for the diagnosis of intrauterine infection is detection of CMV in the amniotic fluid by culture and PCR. One of the first studies observed that amniocentesis correctly identified 12 of 13 (92%) infants with congenital CMV infection [39]. A subsequent study observed that amniocentesis was 100% sensitive in diagnosing congenital CMV infection [40]. A more recent study observed that viral culture of amniocentesis was 77% sensitive in detecting congenital CMV infection and the specificity was 100% [41]. Low sensitivity (false negative results) in some studies is probably due to infants becoming infected *in utero* after the amniotic fluid sampling. False positive results are rare and when they occur may be due to maternal contamination of amniotic fluid. For maximal accuracy, both viral culture and PCR should be obtained. A diagnosis of fetal CMV infection alone is insufficient to predict newborn disease. A large amount of virus as measured by PCR in the amniotic fluid is most likely related to gestational age and should not be used as an independent predictor of a poor fetal outcome [42, 43]. 

## 6. Sensitive Fetal and Placental Indicators for Neonatal Outcomes

Among CMV-infected fetuses, fetal abnormalities or placental enlargement by ultrasound is predictive of newborn disease and a poor long-term outcome [11, 15, 44, 45]. In a prospective study of passive immunization using CMV hyperimmune globulin, any ultrasound abnormality, excluding placental thickening, was after multivariate analysis an independent predictor (*P* < 0.001) of a poor newborn outcome [11]. Numerous ultrasound abnormalities have been described in association with intrauterine CMV infections and because of normal variations and alternative causes care must be taken when basing therapeutic decisions solely on ultrasound abnormalities [11, 44, 45].

One study has evaluated placental thickening in women with primary CMV infections during pregnancy [15]. In that study, the placental size of 93 women with a primary infection and 73 CMV-seropositive pregnant women without primary infection were evaluated. Placental ultrasound evaluations were performed from 16-to-36-week-gestation. Women with primary CMV infection and a fetus or newborn with CMV disease had significantly (*P* < 0.0001) thicker placentas than women with a primary infection whose fetuses or newborns were disease free. Women with a primary infection and whose fetuses were uninfected still had significantly (*P* < 0.0001) thicker placentas than seropositive controls without infected fetuses, suggesting the placentas were infected even though the fetuses were uninfected. Placental thickness values, predictive of primary maternal infection and/or fetal disease, were observed at each biweekly measurement from 16-to-36-week-gestation, and cutoff values ranged from 22 to 35 mm, with the best sensitivity and specificity at 28 and 32 weeks [14]. Other causes of placental thickening were excluded in these patients [15]. 

## 7. Interventions

Both the CDC and ACOG recommend that pregnant women be counseled on ways to reduce their risk of CMV acquisition during pregnancy [46–48]. These simple hygienic precautions are listed in Table 1. Studies demonstrating the efficacy of hygienic precautions are few but compelling. Studies completed by our group demonstrated that these hygienic measures when provided to CMV seronegative pregnant women with a young child in the home were effective [22, 23]. In our studies, women were educated about CMV, and provided written detailed guidelines detailing hygienic precautions in Table 1, watched a video on how to practice these precautions, and then were permitted to ask questions of a research nurse. These precautions were well received by all of the pregnant women and were easily accomplished. Based on interviews and on a written survey done before enrollment and at the end of pregnancy, none of 130 seronegative pregnant women complained the precautions were burdensome or anxiety provoking during pregnancy [22, 23]. Further, none of the pregnant women declined serotesting. To date, of 37 pregnant women with a child shedding CMV, we have observed only one who received hygienic precautions and seroconverted to CMV during pregnancy, compared to infection rates of 42% for 64 of 154 nonpregnant women with a child shedding CMV, including seronegative women who were trying to conceive [22]. 

This observation has recently been confirmed and expanded in a French study where 5312 pregnant women were offered CMV serologic screening during pregnancy [12]. Of these women, 97.4% agreed to screening and signed a consent. If an initial serologic test was negative at 12 weeks gestation, detailed hygienic information was given orally and in writing to the woman and her spouse. Wearing protective gloves was not recommended. For 2595 seronegative women, the rate of maternal seroconversion during the first 12 weeks of gestation was compared to the rate between weeks 12 and 36. Prior to patient education and the receipt of hygienic precautions at 12 weeks, the maternal seroconversion rate was 0.42%, compared with a rate of 0.19% for women from week 12 to 36 of the gestation. When adjusted for the number of woman-weeks observed, the rate prior to 12 weeks gestation was 0.035% per woman-week compared to a rate of 0.008% per woman-week after intervention (*P* = 0.005). Maternal primary infections and seroconversions were distributed evenly throughout gestation [12]. 

These studies provide compelling data on the simplicity and effectiveness of hygienic intervention to prevent CMV infection of high-risk pregnant women who are tested for CMV during pregnancy. Although seronegative health care workers do not have an increased risk, pregnant child care employees are at a significant risk for CMV acquisition. In 2008, the CDC website recommended that pregnant child care employees be informed they could assess, their risk by serologic testing and if seronegative, avoid if possible caring for children less than 2 years age for the duration of pregnancy.

Serial serologic screening would identify pregnant women with a primary CMV infection prior to fetal infection. For these women, prompt passive immunization may prevent fetal infection. Prevention of fetal infection with a high titer CMV immunoglobulin (HIG) preparation was reported in 2005 [11]. In a prospective Phase I-II trial, 181 pregnant women with a primary CMV infection were identified. Most women were asymptomatic and identified by serologic screening. For women with a primary infection at <21 weeks' gestation or for those who refused amniocentesis, HIG (100 U/kg of maternal weight) was administered monthly until delivery. Of 126 women (mean gestational age at infection 14.3. + 7 weeks) who did not receive HIG, 56% delivered infected infants, compared with 16% of 37 women (mean gestational age at infection, 13.2 + 5.5 weeks) who received prophylactic HIG (*P* < 0.001). In this study, it is likely that the true efficacy of HIG may have been greater than actually observed because a few of the fetuses may have been infected *in utero* before HIG was administered. Although this was not a randomized controlled trial, the observations were consistent with the observations for natural infection. That is, women who have prepregnancy antibodies to CMV acquired by a natural infection have a markedly reduced rate of mother-to-fetus transmission of CMV. 

 In the same study, passive immunization was also evaluated in women with proven fetal infection [11]. Forty-five women had a primary infection more than 6 weeks before enrollment and underwent amniocentesis to detect CMV DNA or virus in amniotic fluid. Thirty-one of these women, whose fetuses were infected, received HIG. Fourteen women with infected fetuses declined HIG and half of them delivered infants with a symptomatic CMV infection. In contrast, only 1 of the 31 women who received HIG delivered a diseased infant at birth (adjusted odds ratio, 0.02; *P* < 0.001). In particular, 15 treated women had fetuses with ultrasound abnormalities consistent with an intrauterine CMV infection. Fourteen infants of these 15 fetuses were healthy despite the prenatal ultrasound signs of involvement. Administration of HIG to the mother and fetal ultrasound abnormalities before treatment were independent predictors of fetal outcome (*P* < 0.001). 

After primary infection, for women with or without infected fetuses or newborns, treatment with HIG was associated with significant (*P* < 0.001) reductions in placental thickness, placental inflammation, and placental viral load for seroconversion for gestational weeks 12 to 36 [15, 16]. A limitation of HIG administration is that it does not appear to affect hearing deficit [17]. This is anticipated since the incidence of hearing deficit among congenitally infected infants is independent of preconception high titer high avidity maternal antibodies and may progress postnatally in spite of high titer neonatal antibodies [13]. 

Regarding the safety of HIG, no toxicity has been observed and immunoglobulins have been used safely in pregnancy since the 1950s [47–51]. Intravenous immunoglobulins are the most purified among the blood derivatives, including albumin, and are pasteurized. Intravenous immunoglobulins are used each year to safely treat many tens of thousands of patients such as transplant patients and those with Kawasaki's disease, immune deficiencies, thrombocytopenia, and so forth. For a woman with a primary CMV infection during pregnancy, legitimate safety concerns have to be weighed against the risk of an affected infant or fetus. 

## 8. Cost Benefit and Feasibility of Maternal Screening

 Given that nine countries routinely screen half or more of all pregnant women serially during pregnancy for seroconversion to CMV, it appears that the logistical and economic challenges of implementing screening for CMV on a large scale have been overcome in these countries. In the USA pregnant women are now routinely screened for rubella, syphilis, hepatitis, and HIV. Thus, adding CMV to ongoing serologic testing is feasible. 

The cost/effectiveness of both passive and active immunization against CMV have been formally estimated in 4 studies and was cost-effective in each study [4, 52–54]. Two cost/benefit studies have addressed universal screening and passive immunization [53, 54]. One recent study found that universal serologic screening (as compared to screening, only high-risk women or those with abnormal ultrasound findings) was the preferred and most cost-effective approach [54]. This conclusion assumed that serologic testing occurred one time at 20 weeks gestation and that passive immunization as therapy (as opposed to prevention) would be at least 47% effective. Another recent study of passive immunization during pregnancy also used QUALYS and decision analysis (CEA ) [53]. This model also incorporated universal maternal serial serological screening in pregnancy. In both the treatment and prophylaxis protocols as described by Nigro et al. [11], universal maternal serologic screening for CMV status and seroconversion in pregnancy followed by maternal HIG was cost-effective in the reduction of CMV sequelae in newborns. The model demonstrated both cost savings and a reduction in CMV disease cases. The optimal model demonstrated a reduction in congenital CMV cases with disease from 7.2/10,000 to 3.5/10,000 requiring treatment of 2.3 pregnancies per case prevented. This model applied over the majority of the US population, remaining cost effective for maternal seroconversion rates in pregnancy >1%, termination rates <10%, and maternal prepregnancy immunity rates of 50–80%.

## 9. Comment

Universal serologic screening whether by an initial blood test or by serial testing during pregnancy for a primary CMV infection is controversial [55, 56]. In the USA, all women are screened via ultrasound at around 20 weeks gestation. When CMV-associated fetal abnormalities are detected and an intrauterine CMV infection confirmed by amniocentesis, data from the new National CMV Registry for Pregnant Women (CMVregistry.org) indicate off-label HIG is often used. Of the first 48 women enrolled in the registry, 23 had positive amniotic fluid and 18 (78%) were treated off-label with HIG (Cytogam). While apparently safe, this approach is far from ideal. Treatment is given only to fetuses of mothers infected in early gestation and who have the poorest prognosis. 

Although some have argued for not using HIG without a randomized clinical efficacy trial, for several reasons such a trial is very unlikely and no such clinical trial is ongoing or contemplated [56, 57]. Obstacles to a therapeutic trial include: subject acceptance of placebo when the study drug appears safe and is readily available off label, the need to serologically test over 100,000 pregnant women to identify a few hundred with fetal infection, the high costs associated with a large multicenter trial and a lack of interest by government, and industry in financing such a trial. 

A better clinical trial design is one that prevents fetal infection with HIG following a primary maternal infection. Two such trials are ongoing in Europe where serologic screening identifies CMV-infected women during pregnancy. The interim results of one such trial are encouraging [58]. To perform these studies in the USA requires annual serologic screening of at least 20,000 pregnant women and to date no such studies have been funded in the USA. 

Postponing CMV testing of pregnant women until the problem is solved by an active vaccine is unrealistic. Older active CMV vaccines have had limited success and there are no current clinical trials of new vaccines [59, 60]. Thus the licensing of an active CMV vaccine is probably at least a decade away. 

Hygienic intervention for high-risk women is appropriate now. At least one-third of the pregnant women in the US are high risk (Table 2); that is, they have daily household or occupational contact with children less than 3 years old. Offering an initial blood test for CMV IgG antibodies in pregnancy, educating them about CMV, and providing simple hygienic precautions could be routine. Testing of their living children for CMV excretion is not useful since they may not be shedding CMV initially but start shedding at anytime during their mother's pregnancy [22]. 

**Figure 2:**
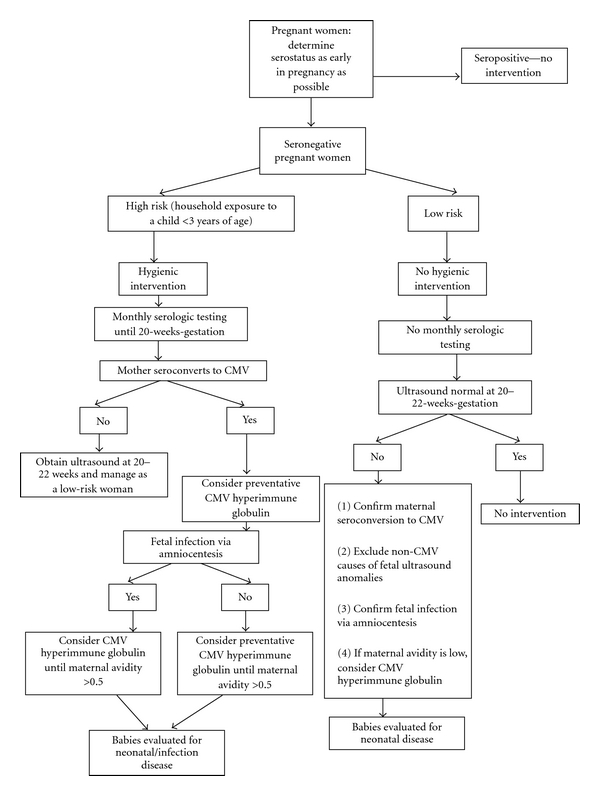
A possible algorithm for limited CMV screening during pregnancy.

Whether to continue serial testing for seroconversion during pregnancy could initially be a decision for each woman and her obstetrician. In other countries the main rationale for universal serial screening was apparently to allow for an elective termination of pregnancy. In Israel approximately half of women who seroconvert to CMV electively terminate, although this rate is much lower in other countries [61, 62]. Figure 2 shows one possible algorithm for limited maternal screening. 

An alternative to serologic screening is to provide all high-risk pregnant women the hygienic interventions. In the reported studies, however, women knew their serologic status, so it is unclear if a pregnant woman's perception of her risk (susceptible) would affect efficacy. Hygienic precautions do not work in nonpregnant women, suggesting seronegative pregnant women perceive a high risk and are more motivated to comply [22, 23]. It is very likely that most high-risk women would want to assess their risk by knowing their serologic status given that serologic testing is readily available. 

Even limited serologic screening as suggested in Figure 2 has potential adverse effects such as false positive or negative IgG results which may lead to apparent seroconversion and thus increased costs associated with additional serologic testing or unnecessary imaging and amniocentesis. The negative impact of these potential problems has not been reported, although this could be studied in countries that now use routine serologic screening. 

Regardless of the strategy used, no serologic testing, only hygienic precautions, one time testing, or serial serologic testing through out the first two trimesters of pregnancy, education of pregnant women about CMV is necessary. With a rare exception, the reaction of women and especially those who acquire a CMV infection during pregnancy is that they wish they had known about CMV and could have taken some measures to avoid infection. 

## Figures and Tables

**Table 1:** Practices for seronegative pregnant women to reduce risk of CMV infection.^#^

(1) Assume that children under age 3 years in your care have CMV in their urine and saliva	
(2) Thoroughly wash hands with soap and warm water after:	
diaper changes and handling child's dirty laundry	
feeding or bathing child	
wiping child's runny nose or drool	
handling child's toys, pacifiers, or toothbrushes	
(3) Do not:	
share cups, plates, utensils, toothbrushes, or food	
kiss your child on or near the mouth	
share towels or washcloths with your child	
sleep in the same bed with your child	

^#^From [22].

**Table 2:** Estimated annual impact in the USA of group child care on the rate of congenital CMV infection among multiparous Caucasians compared to multiparous non-Caucasians without group child care.

	Caucasian	Non-Caucasian
No. live births/year	3,000,000*	1,000,000*
No. of seronegative mothers at conception	1,800,000 (60%)	200,000 (20%)^&^
No. with previous child at home	900,000 (50%)	Unknown
No. with previous child <3 years at conception	675,000 (75%)	Unknown
No. with child in day care	506,250 (75%)	Unknown
No. with shedding child	126,563 (25%)	Unknown
No. becoming infected during pregnancy	53,156 (42%)	15,600 (7.8%)^&^
No. of infants infected *in utero* (50%)	26,578	7,800
No. of infants with severe sequella^+^ (28%)	7,442	2,184

*US Bureau of Vital Statistics.

^+^Death, I.Q. < 70, or deafness.

^&^Based on data from [3, 13, 14].
